# Evaluating the diagnostic performance of six plasma biomarkers for Alzheimer’s disease and other neurodegenerative dementias in a large Chinese cohort

**DOI:** 10.1186/s13195-025-01712-y

**Published:** 2025-04-03

**Authors:** Bin Jiao, Ziyu Ouyang, Yiliang Liu, Cong Zhang, Tianyan Xu, Qijie Yang, Sizhe Zhang, Yuan Zhu, Meidan Wan, Xuewen Xiao, Xixi Liu, Yafang Zhou, Xinxin Liao, Weiwei Zhang, Shilin Luo, Beisha Tang, Lu Shen

**Affiliations:** 1 Department of Neurology, Xiangya Hospital, Central South University, Changsha, 410008 China; 2 National Clinical Research Center for Geriatric Disorders, Xiangya Hospital, Central South University, Changsha, 410008 China; 3 Hunan International Scientific and Technological Cooperation Base of Neurodegenerative and Neurogenetic Diseases, Xiangya Hospital, Central South University, Changsha, 410008 China; 4 Engineering Research Center of Hunan Province in Cognitive Impairment Disorders, Central South University, Changsha, 410008 China; 5 Key Laboratory of Hunan Province in Neurodegenerative Disorders, Central South University, Changsha, 410008 China; 6 Department of Geriatrics, Xiangya Hospital, Central South University, Changsha, 410008 China; 7 Department of Radiology, Xiangya Hospital, Central South University, Changsha, 410008 China; 8 Brain Research Center, Central South University, Changsha, Hunan 410008 China

**Keywords:** Alzheimer’s disease, Plasma biomarker, Phosphorylated-tau, Glial fibrillary acidic protein, Neurofilament light chain, α-synuclein

## Abstract

**Background:**

Ethnic variations and detection methods may lead to differences in diagnostic biomarkers of dementia, and few comparative studies have evaluated the six plasma biomarkers of Alzheimer’s disease (AD) and other neurodegenerative dementias in the Chinese population.

**Methods:**

A cross-sectional cohort of 668 participants were enrolled, including 245 amnesic mild cognitive impairment (aMCI) or AD patients with Aβ positive pathology, 67 with frontotemporal dementia (FTD), 100 with progressive supranuclear palsy (PSP), 72 with dementia with Lewy bodies (DLB) and 184 healthy controls. Additionally, a longitudinal subset of 19 aMCI and 30 AD patients was followed for an average period of 1 year. Plasma biomarkers, including p-tau181, p-tau217, p-tau231, NfL, GFAP, and α-synuclein, were simultaneously measured using a novel single molecular array method. Aβ42 and p-tau181 levels in CSF, amyloid PET and structural MRI were measured.

**Results:**

Plasma p-tau217 and p-tau231 were most effective in diagnosing aMCI/AD (AUC = 0.95 and 0.93, respectively), while p-tau217, p-tau231 and p-tau181 presented the best differential diagnosis for AD from PSP, FTD and DLB respectively (AUC = 0.84, 0.81 and 0.83). α-synuclein was presented as the best biomarker for PSP variant and behavior variant FTD subtypes (AUC = 0.81 and 0.74, respectively). Among them, p-tau217, p-tau231, GFAP and a-synuclein were negatively correlated with CSF Aβ42/40, while p-tau217 and GFAP were positively correlated with CSF p-tau181. Besides, p-tau181, p-tau217, and GFAP were associated with temporal lobe volume, while p-tau231 and GFAP were associated with frontal lobe volume. Longitudinal analysis showed the higher p-tau181 could predict the cognitive decline progression.

**Conclusions:**

This study validate the practicality of blood biomarkers in the Chinese Han population using a novel single molecule immune detection method. Through the clinical performance study for several biomarkers, we found the plasma p-tau217 was the most effective biomarker in AD diagnosis, and p-tau showed high accuracy for differential diagnosis of AD from other dementia, GFAP is associated with multiple aspects of AD pathology, and frontal and temporal lobe volume, and p-tau181 can reflect the dynamic cognitive decline of AD.

**Supplementary Information:**

The online version contains supplementary material available at 10.1186/s13195-025-01712-y.

## Background

Alzheimer’s disease (AD) is the most common cause of dementia, affecting tens of millions of people worldwide [1]. Currently, the core markers of AD are abnormal amyloid beta (Aβ) and tau aggregation detected by positron emission tomography (PET), as well as Aβ42 and phosphorylated tau (p-tau) levels in cerebrospinal fluid (CSF) [2]. These biomarkers reflect the neuropathology of AD and begin to accumulate two decades before the appearance of clinical symptoms [3]. Although Aβ and tau biomarkers from PET and CSF are highly accurate in detecting AD pathology, their high cost, invasiveness, and low availability hinder their feasibility in clinical practice.

Recent technological advances have enabled the measurement of plasma biomarkers with great potential for AD detection. Several studies have shown that plasma p-tau isoforms, such as p-tau181, p-tau217, and p-tau231, are highly accurate and specific for the detection of PET-confirmed Aβ and tau pathology across the clinical AD continuum in European and American populations; thus, these are recognized as the most promising plasma biomarkers in detecting AD [4–6]. Moreover, plasma p-tau has strong value in differential diagnosis, with high accuracy for discriminating patients with AD from those with frontotemporal dementia (FTD) [7, 8], progressive supranuclear palsy (PSP) [9, 10] and dementia with Lewy bodies (DLB) [11, 12]. However, the optimal method for detecting AD remains controversial. Janelidze S et al. conducted a head-to-head comparative study and found that p-tau217 performed best when identifying patients with mild cognitive impairment (MCI) with abnormal brain Aβ or those who will subsequently progress to AD [13]. Moreover, Ashton NJ et al. reported that p-tau231 showed the greatest potential to detect AD pathology in the earliest disease stages [6]. Longitudinal studies in diverse ethnic groups have shown that plasma biomarkers are associated with the progression of AD [2, 14, 15], among which longitudinally increased p-tau217 was found to be associated with cognitive impairment and brain atrophy in preclinical AD [14]. Other biomarkers, which are non-specific, but highly associated with disease progression biomarkers, such as neurofibrillary light chain (NfL) [16] and glial fibrillary acidic protein (GFAP) [17], have also been shown to contribute to AD diagnosis. Additionally, α-synuclein, the main component protein of Lewy bodies, has been found in the brains of patients with AD, indicating its role in the pathophysiology of AD [18]; however, the diagnosis efficiency of plasma α-synuclein for AD remains unclear. Considering that these biomarkers may be influenced by racial disparities, and given the lack of systematic study based on the Chinese population, it is necessary to conduct a comparative study for several biomarkers in this population.

Currently, various methods for biomarker detection have been reported to have good performance, including immunomagnetic reduction (IMR), enzyme-linked immunosorbent assay (ELISA), single molecule immune detection (SMID) [13, 19–21] et al. Single-molecule assay (SIMOA) is currently the most representative single-molecule immunoassay technology. In China, Astra System is a patented novel single molecule immune detection system based on single nano-fluorophore imaging, with the advantages of less consumables, low cost and fast detection speed, which has been applied to several studies for fluid molecules detection [22–25]. However, no previous study has evaluated the performance of plasma biomarkers using Astra System in a large Chinese dementia cohort. Therefore, we first used Single Molecular Immunity Detection kits (Astrabio, Suzhou, China) to simultaneously detect the six plasma biomarkers to explore whether their diagnostic performance is consistent with previously reported results. In general, the aims of this study can be summarized as follows: (1) To compare the levels of six plasma biomarkers, including p-tau181, p-tau217, p-tau231, NfL, GFAP, and α-synuclein, simultaneously in a Chinese cohort of 668 participants, including MCI, AD, FTD, DLB, and PSP, and healthy controls (HCs); (2) to calculate the diagnostic and differential diagnostic performance of plasma biomarkers for AD; (3) to clarify the associations of biomarkers with AD pathology; and (4) to evaluate the relations between plasma biomarkers and gray matter volume, as well as baseline and subsequent longitudinal cognitive decline.

## Methods

### Participants

This cross-sectional study enrolled 668 participants, including 54 with amnesic MCI (aMCI), 191 with AD, 100 with PSP, 67 with FTD, 72 with DLB, and 184 HCs in the Department of Neurology, Xiangya Hospital, Central South University. Among them, 49 patients with aMCI or AD completed a longitudinal analysis. AD and aMCI were diagnosed based on the National Institute on Aging and Alzheimer’s Association criteria (2011) [26], FTD, DLB, and PSP were diagnosed according to their respective clinical criteria [27–30]. Among them, 237 patients completed PiB-PET or CSF biomarkers (Aβ42, Aβ40, t-tau, and p-tau181) detection and were divided into Aβ positive(+) & Aβ negative(-), tau positive(+) & tau negative(-) groups according to the 2018 NIA-AA criteria [31]. PSP was further subdivided into Richardson’s syndrome (PSP-RS) and PSP variant (PSP-v) according to international criteria [30]. FTD was further subdivided into behavioral variant FTD (bvFTD), non-fluent variant FTD (nfvFTD), and semantic variant FTD (svFTD) [32]. All HCs were recruited from the Liuyang community and showed no cognitive decline after 2 years of follow-up.

### Neuropsychological assessment

All patients underwent a comprehensive battery of neuropsychological tests, including the Mini Mental State Examination (MMSE), Montreal Cognitive Assessment (MoCA), and Clinical Dementia Rating (CDR) assessment. All HCs completed the MMSE assessments at baseline and the 1-year follow-up respectively.

### Magnetic resonance imaging (MRI)

T1-weighted MRI was performed using a 3T MRI scanner (Prisma, Siemens, Germany) with a 64-channel head/neck coil, using a magnetization prepared rapid gradient echo (MP-RAGE) sequence with the following parameters: repetition time, TR = 2,300 ms; echo time (TE) = 2.98 ms, 1 mm isotropic voxel size and 176 slices. The CAT12 toolbox (http://dbm.neuro.uni-jena.de/cat/) implemented in SPM12 running in MATLAB was used to estimate cortical thickness and grey matter volume. All images were checked with an internal quality assurance protocol following CAT12-recommended values/indicators, segmented into gray matter (GM), white matter (WM), and CSF, and normalized using the DARTEL algorithm. For smoothing, we applied an isotropic Gaussian kernel of 10 mm full width half maximum (FWHM).

### PiB-PET acquisition and interpretation

^11^C-labelled Pittsburgh compound B (PiB) PET imaging was performed and analyzed as previously described [33]. Briefly, images were acquired 50 min after PiB was injected intravenously (12 mCi, 1 Ci/µmol, over 20 s). The examinations were performed by qualified nuclear medicine technologists. Cortical regions exhibiting the most distinct radiotracer accumulation in Aβ-positive subjects typically include lateral temporal, frontal lobes, posterior cingulate cortex/precuneus, and the parietal lobes, whereas the sensorimotor cortex and the visual cortex can be relatively spared [34]. The cerebellum was used as the reference region for visual interpretation. The results were interpreted by three experienced nuclear medicine physicians who completed the appropriate training programs provided by the radiotracer manufacturer. Standardized uptake value ratios (SUVR) were calculated as the ratio of regional PiB retention to that in the cerebellar gray matter. ROIs were drawn on the lateral temporal, frontal lobes, posterior cingulate cortex/precuneus, and the parietal lobes. Individuals with SUVR values exceeding the cutoff point of 1.5 in any of these ROIs was classified as Aβ(+).

### Cerebrospinal fluid collection and analysis

CSF was collected via lumbar puncture, before centrifuging at 2000 × g, 4℃for 10 min, and stored at -80℃. All CSF biomarkers were measured by ELISA, including beta-amyloid (1–40) (Aβ40) (Eqs. 6511-9601-L), beta-amyloid (1–42) (Aβ42) (Eqs. 6521-9601-L), total-tau (t-tau) (Eqs. 6531-9601-L), and phosphorylated-tau181 (p-tau181) (Eqs. 6591-9601-L) (EUROIMUN, Germany). According to the manufacturer’s instructions, participants with Aβ42 < 550 pg/ml or Aβ42: 551–650 pg/ml along with Aβ42/40 ≤ 0.1 were classified as Aβ-positive (A+), and participants with p-tau ≥ 61 pg/ml were classified as tau-positive (T+).

### APOE genotyping

Genomic (gDNA) was isolated from peripheral blood using the standard phenol-chloroform extraction method. All gDNA samples were diluted to 50 ng/µl. The following primers were used to amplify the 581 bp fragment: forward: 5’-CCTACAAATCGGAACTGG-3’; and reverse: 5’-CTCGAACCAGCTCTTGAG-3’. Polymerase chain reaction (PCR) was performed as described previously. The PCR products were sequenced using an ABI 3730 DNA Analyzer (ABI, Louis, MO, USA).

### Sample collection and processing

Venous blood of participants was collected in ethylenediaminetetraacetic acid (EDTA) tubes and centrifuged at 3000 rpm for 15 min at 4℃ within 2 h of collection. The obtained blood samples for plasma were stored at − 80℃, and none of the samples underwent freeze-thaw cycles prior to their use in the experiments.

### SMID detection

P-tau181, p-tau217, p-tau231, NfL, GFAP, and α-synuclein were quantified on a fully automated machine (AST-Sc-Lite; AstraBio, Suzhou, China). A detailed description of SMID in the Astra System, including a schematic illustration, standard curves, limit of detection (LOD), and limit of quantitation (LOQ) for measuring plasma biomarkers, as well as an assay comparison between AST-Sc-Lite and Quanterix/Mesoscale Discovery, is provided in Supplementary Figs. 1–5, Supplementary Tables 1–3). The working process of SMID in Astra System is as follows: (1) 25 µL Reagent 1 solution containing 1.2 μm magnetic beads obtained from Merck (EM1-100/40) bond with capture antibody was first mixed with 25 µL sample, followed by 6 min incubation under 40℃. The capture antibody chemically modified on magnetic beads bond to antigen in sample solution. (2) 10 µL Reagent 2 solution containing detection antibody labelled nano-fluorophore was then added. After a quick mix, another 4 min incubation under 40℃ happened to let detection antibody labelled with fluorophore can bind to antigen capture by magnetic beads. (3) The mixture was then moved to a flow-cell (Supplementary Fig. 6). A permanent magnet was pressed onto bottom of flow-cell to gathering magnetic beads straightly down onto bottom surface in the flow-cell channel, which was sized as 2 mm*2 mm*60 mm (height*width*length). Due to a well control of verticality of magnetic field direction to channel surface and high uniformity of magnetic beads distributed in the mixture solution, magnetic beads generated a homogeneous but random single-layer array on the channel bottom surface. By controlling of magnetic beads concentration, agglomeration of beads to beads was strictly avoided, while a rare probability of beads may still gather with no effecting on detection variation. Contributed by a fully automated control, the variation of beads collected on the surface was no more than 1.2% between each measurement. (4) Afterwards, unbonded fluorophore was washed away and fluorescent image of beads array was taken by an integrated camara with excitation light source (Supplementary Fig. 7). (5) The counting number was further used for antigen quantitation. Variation of magnetic beads number gathered in generated single-layer beads array contribute to variation of measuring result directly. Thus, a stable control of beads number is essential. We estimated the stability of beads array under different measurements. 1.2% CV% of beads number for 10 repeats indicated a well-controlled beads array generation approach for further quantitation application. P-tau181 (R64030), p-tau217 (R64100), p-tau231 (R64050), NfL (R64040), GFAP (R64060), and α-synuclein (R64070) assay kits (AstraBio) were used for detection. The technicians were blinded to the group status of the participants.

### Statistical analysis

Statistical analyses were performed using the R software (version 4.2.3). Differences in plasma biomarkers between groups were determined using a univariate general linear model (GLM). The Bonferroni correction was used for multiple comparisons. FC was used to describe the difference between two groups. The differential analysis focused on the differences in the distribution of plasma biomarkers between the HC and disease groups, between AD and other dementias, between different pathologies, between degrees of severity, and between the HC and different PSP and FTD subtypes. Classification analysis was performed using receiver operating characteristic (ROC) curve analysis to estimate the diagnostic ability of the plasma biomarkers using the R package cutpointr (https://github.com/thie1e/cutpointr) calculating a cut-off score using the Youden’s index and the area under the curve (AUC) was reported for each comparison. The R package RISCA (https://github.com/cran/RISCA) was used to visualize ROC curves. Classification analysis focused on the ability of plasma markers to distinguish aMCI and AD from HC, DLB, FTD, and PSP, as well as distinguish the HC from different PSP and FTD subtypes. Spearman’s test was used to evaluate the correlation between the plasma and cerebrospinal fluid markers. For voxel-based analysis of gray matter volume and blood biomarker concentrations, segmentation and smoothing of the brain model were performed using CAT12. The MRI data were spatially smoothed using an 8 × 8 × 8 mm FWHM Gaussian kernel. Correlations were assessed by GLM analysis using SPM12. To study associations of longitudinal changes in plasma biomarkers with longitudinal cognition, we used linear mixed effects models with the interaction between time and standardized plasma biomarker slopes (derived from subject-level linear regression models, with time as predictor of biomarker levels) as the independent variable. The R package visreg (https://github.com/cran/visreg) was used to visualization. Sex, age, and *APOE ε4* genotype were included as covariates in the above analysis. The total intracranial volume (TIV) was additionally included as a covariate in gray matter volume analysis.

## Results

### Participant characteristics

In this study, we used cross-sectional and longitudinal analyses to investigate AD biomarkers in the Chinese Han population. The cross-sectional cohort included a total of 668 individuals, while in the longitudinal study, a subset of 49 A + T + N + patients with AD or aMCI (male: 46.9%, age at onset: 68.9 ± 8.6 years, CSF Aβ42: 358.2 ± 140.8 pg/ml, CSF Aβ40: 7907.9 ± 3814.8 pg/ml, CSF Aβ42/40: 0.05 ± 0.01 CSF t-tau: 144.2 ± 29.4 pg/ml, CSF p-tau: 567.3 ± 212.2 pg/ml) underwent cognitive assessments and plasma biomarker detection after an average of 1-year period (1.2 ± 0.6 years). The baseline demographics, cognitive assessment scores, and CSF biomarker levels are shown in Table 1. There was no significant difference in sex (*p* < 0.17), whereas the AD + aMCI patients were younger than HC (*p* < 0.001). Additionally, there was a higher proportion of *APOE* E4 carriers (43%) among the AD + aMCI group than the HC group (17%, *p* < 0.001).

**Table 1:** Demographic and clinical characteristics of the participants in the cross-section cohort

Characteristic	AD + aMCI (*n* = 245)	PSP (*n* = 100)	FTD (*n* = 67)	DLB (*n* = 72)	HC (*n* = 184)	*p*-value^1^
Sex (Male, %)	40%	61%	60%	60%	46%	0.17
Age of onset (Years)	63.1 ± 9.8	65.2 ± 6.9	61.9 ± 9.5	71.4 ± 9.1	67.6 ± 6.5	< 0.001
*APOE E4* ^*2*^	43%	15%	31%	36%	17%	< 0.001
Education (Years)	9.2 ± 3.6	8.7 ± 3.3	9.2 ± 3.4	8.3 ± 3.7	8.1 ± 2.0	< 0.001
MMSE	16.4 ± 8.4	22.0 ± 6.4	15.9 ± 9.2	14.2 ± 7.4	28.9 ± 0.9	< 0.001
MoCA	11.3 ± 6.8	14.0 ± 6.4	11.0 ± 7.2	7.9 ± 6.0		
CDR	1.1 ± 0.1	/	1.3 ± 0.1	1.4 ± 0.1		
CSF Aβ42 (pg/ml)	392.3 ± 230.0	1,292.2 ± 772.1	757.4 ± 389.6	506.5 ± 536.2		
CSF Aβ40 (pg/ml)	6,566.6 ± 4,219.4	10,982.8 ± 7,062.0	7,618.5 ± 6,280.7	7,604.6 ± 6,423.5		
CSF Aβ42/40	0.07 ± 0.04	0.1 ± 0.04	0.1 ± 0.1	0.09 ± 0.07		
CSF t-tau (pg/ml)	397.6 ± 268.5	225.4 ± 81.5	225.0 ± 162.0	282.3 ± 161.6		
CSF p-tau (pg/ml)	94.0 ± 53.2	27.0 ± 13.6	25.6 ± 12.6	55.2 ± 33.8		

^1^*p* values were compared between AD + aMCI and HC using Pearson’s Chi-squared test and Wilcoxon rank sum test; ^2^*APOE E4* was defined as *ε3ε4* or *ε4ε4*

### Plasma biomarkers per diagnostic group

**Fig. 1:**
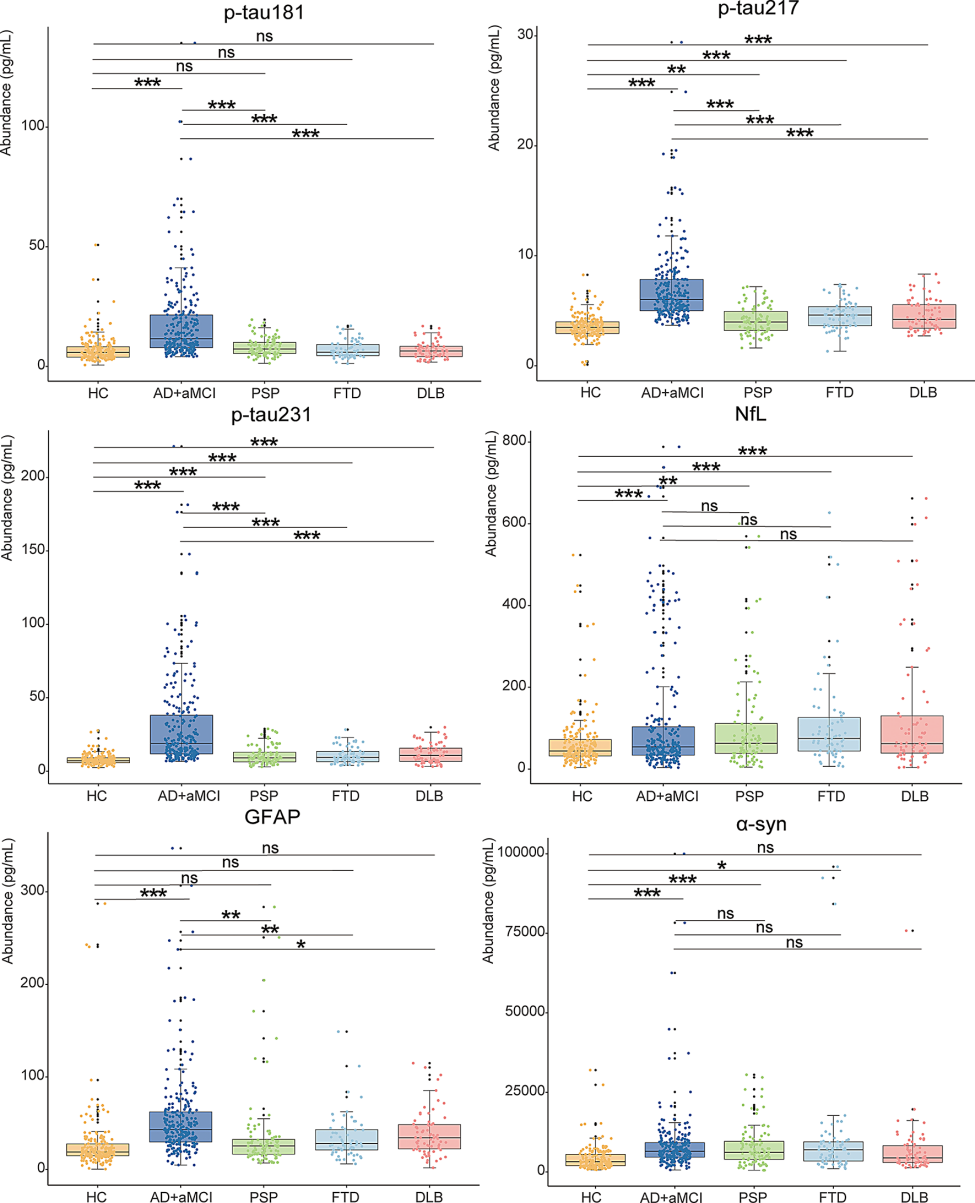
Comparison of blood biomarker levels across different diagnostic groups. The box plots depict the median (horizontal bar) and interquartile range (IQR, hinges). *P*-values were assessed by Wilcox-test. *p < 0.05, **p < 0.01, ***p < 0.001

The levels of plasma p-tau181 (FC = 2.5, *β* = 10.6, *p* < 0.001), p-tau217 (FC = 1.9, *β* = 3.4, *p* < 0.001), p-tau231 (FC = 3.8, *β* = 23.9, *p* < 0.001), NfL (FC = 1.8, *β* = 63.0, *p* < 0.001), GFAP (FC = 2.1, *β* = 29.6, *p* < 0.001), and α-synuclein (FC = 2.0, *β* = 4053.3, *p* < 0.001) were significantly higher in the aMCI/AD group compared to the HC group. When compared to other dementias, the levels of p-tau181 (FC = 2.2–2.6, *β* = 9.4–11.0, *p* < 0.001), p-tau217 (FC = 1.5–1.7, *β* = 2.3–2.9, *p* < 0.001), p-tau231 (FC = 2.5–2.9, *β* = 19.4–20.7, *p* < 0.001), and GFAP (FC = 1.4–1.6, *β* = 17.8–20.3, *p* = 0.002–0.03) were significantly higher in the aMCI/AD group than in other dementias. Besides, the levels of p-tau217 (FC = 1.2–1.3, *β* = 0.5–1.1, *p* < 0.002), p-tau231 (FC = 1.4–1.5, *β* = 2.7–4.3, *p* < 0.001), and NfL (FC = 1.6–2.1, *β* = 41.7–65.9, *p* < 0.003) were increased in other dementias compared to those in the HC group (Fig. 1).

### Classification analyses

**Fig. 2:**
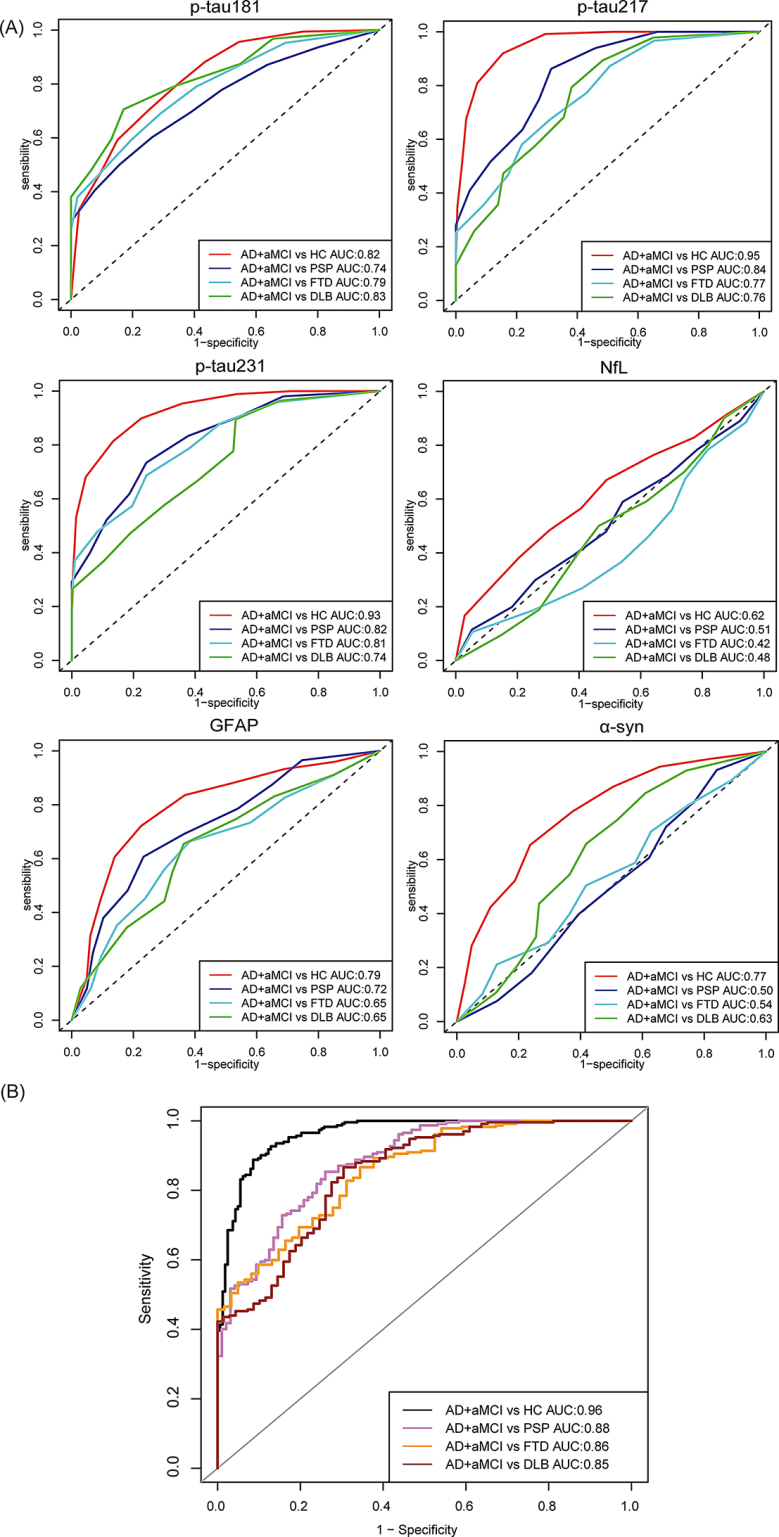
The diagnostic and differential diagnostic performance of single plasma biomarkers and combination of six plasma biomarkers for aMCI/AD using area under the curve (AUC). (**A**) Diagnostic and differential diagnostic performance of single plasma biomarkers; (**B**) Diagnostic performance of the logistic regression (LR) model combining six plasma biomarkers for aMCI/AD diagnosis and differential diagnosis from other neurodegenerative diseases

The levels of p-tau217 and p-tau231 showed the best performance for detecting aMCI /AD from HCs at cut-off values of 4.2 pg/mL and 10.6 pg/mL, achieving AUCs of 0.95 and 0.93, respectively, followed by the levels of p-tau181 and GFAP at cut-off values of 8.7 pg/mL and 24.4 pg/mL, with an AUC of 0.82 and 0.79, and α-synuclein at cut-off value of 3981.6 pg/mL, with an AUC of 0.77. Additionally, the level of NfL at cut-off value of 104.2 pg/mL showed lower power for aMCI/AD diagnosis, with AUCs of 0.62. Interestingly, we found that p-tau217 was the best for detecting aMCI/AD from PSP at cut-off value of 4.5 pg/mL, with an AUC of 0.84; p-tau231 was the best for detecting aMCI/AD from FTD at cut-off value of 10.6 pg/mL, with an AUC of 0.81; and p-tau181 was the best for detecting aMCI/AD from DLB at cut-off value of 8.7 pg/mL, with an AUC of 0.83 (Fig. 2A). Additionally, we compared the diagnostic performance of the logistic regression (LR) model combining six biomarkers, which showed an AUC of 0.96 for aMCI/AD diagnosis. The AUCs for distinguishing aMCI/AD from PSP, FTD and DLB were 0.88, 0.86, and 0.85, respectively (Fig. 2B).

### Associations of plasma biomarkers with AD pathology

**Fig. 3:**
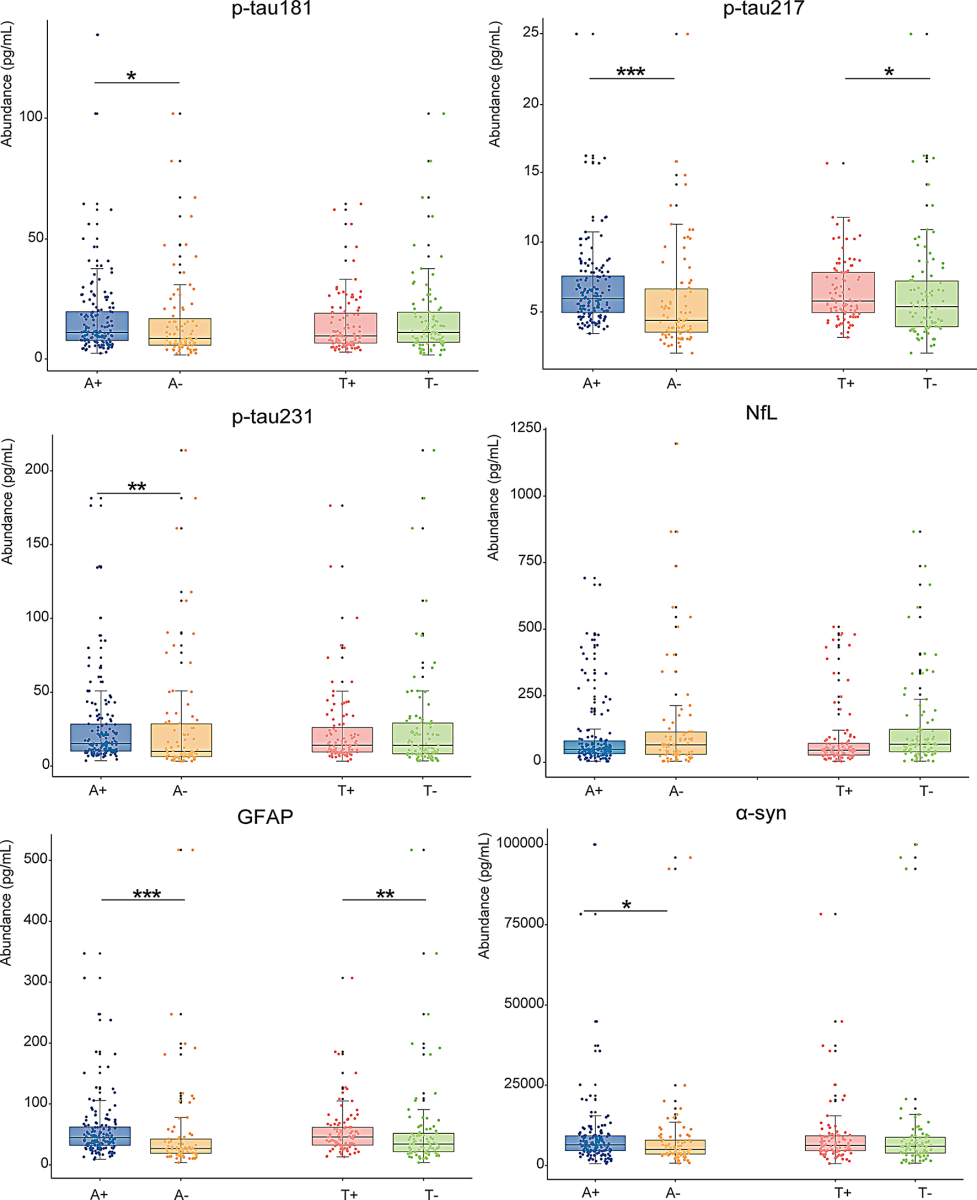
Plasma biomarkers in Αβ-positive versus Aβ-negative dementia group and tau-positive versus tau-negative dementia group. The box plots depict the median (horizontal bar) and interquartile range (IQR, hinges). *P*-values were assessed by Wilcox-test. *p < 0.05, **p < 0.01, ***p < 0.001

**Fig. 4:**
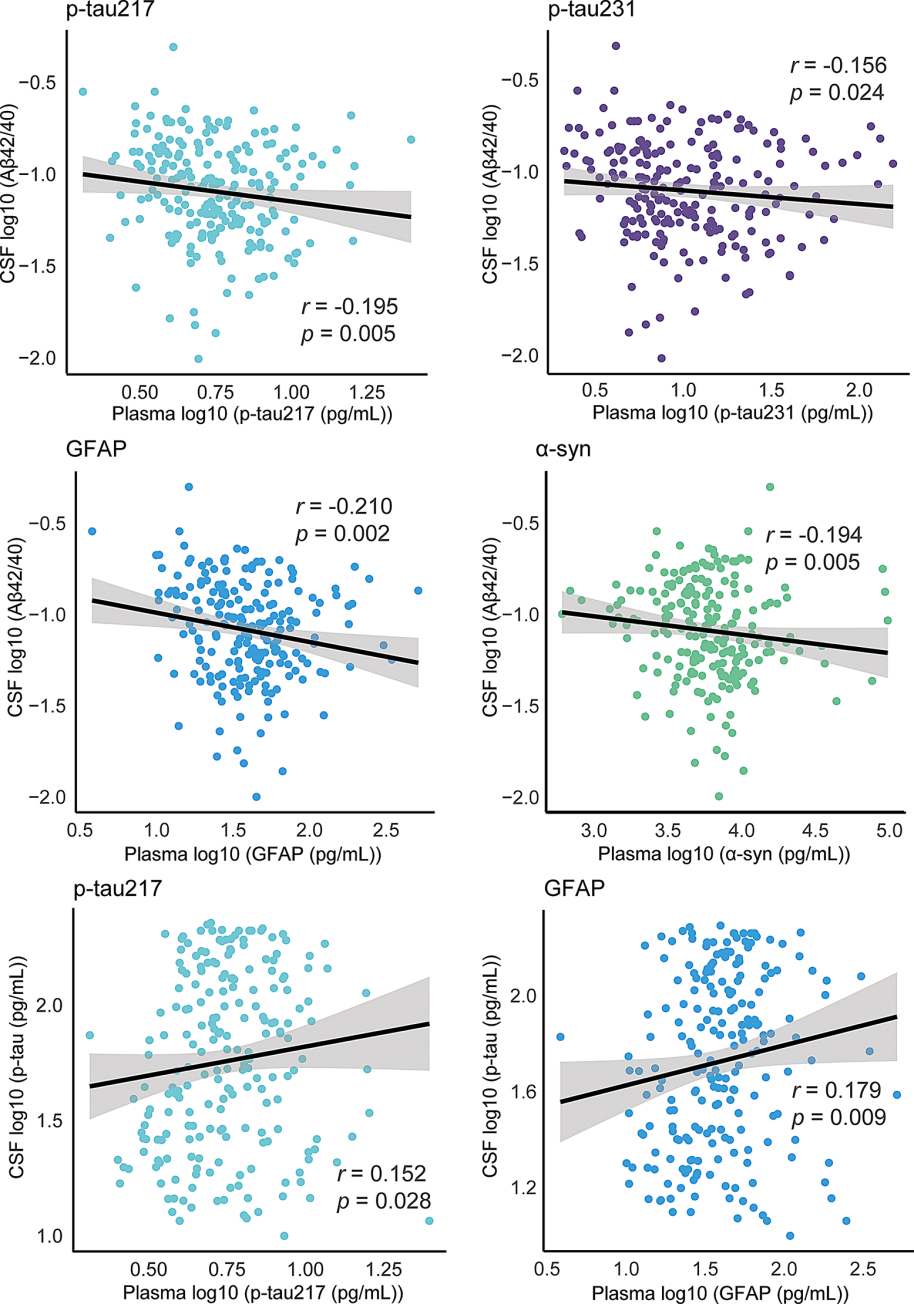
Relationship between plasma biomarkers and CSF biomarkers. P-values were assessed by Spearman’s correlation

We compared the plasma biomarkers in the Aβ (+) (*n* = 153) and Aβ (–) (*n* = 84) groups, as well as the tau (+) (*n* = 100) and tau (–) (*n* = 91) groups, and found that the Aβ (+) group showed higher levels of p-tau181 (*p* = 0.045), p-tau217 (*p* < 0.001), p-tau231 (*p* = 0.002), GFAP (*p* < 0.001), and α-synuclein (*p* = 0.035) than the Aβ (–) group. Furthermore, compared to patients with tau (–) dementia, those with tau (+) dementia had higher levels of p-tau217 (*p* = 0.043), and GFAP (*p* = 0.004) (Fig. 3). We also found that p-tau217, p-tau231, GFAP, and α-synuclein were negatively correlated with CSF Aβ42/40 (*r* = − 0.2, *p* = 0.005; *r* = − 0.2, *p* = 0.024; *r* = − 0.2, *p* = 0.002; *r* = -0.2, *p* = 0.005, respectively), while p-tau217 and GFAP were positively correlated with CSF p-tau181 (*r* = 0.2, *p* = 0.028; *r* = 0.2, *p* = 0.009, respectively) (Fig. 4). We further compared the distribution of plasma biomarkers between A + T+ (*n* = 91) and A + T- (*n* = 40) patients within aMCI/AD group, as well as among the A + T+ (*n* = 5), A + T− (*n* = 13), A − T+ (*n* = 3), and A − T− (*n* = 20) patients within the non-AD (FTD and DLB) groups. The results showed that plasma biomarker levels showed no significant difference between A + T + and A + T- within aMCI/AD group. We also found that in non-AD individuals, NfL levels in the A + T + group were elevated compared to the A − T− group (*β* = 178, *p* = 0.009); p-tau217 and GFAP levels were higher in the A + T − group compared to the A − T− group (*β* = 1.1, *p* = 0.026; *β* = 34.1, *p* = 0.003, respectively); and p-tau217 levels were increased in the A − T + group compared to the A − T− group (*β* = 1.9, *p* = 0.015) (Supplementary Figs. 8 and 9).

### Plasma biomarkers in patients with dementia with different levels of cognitive impairment

Compared to patients with aMCI and mild dementia (CDR = 0.5–1), those with moderate-to-severe dementia (CDR = 2–3) had higher levels of NfL (*p* = 0.013) and GFAP (*p* = 0.020). P-tau181, p-tau217, p-tau231 and α-synuclein showed no significant differences among the different groups (Supplementary Fig. 10).

### Plasma biomarkers and classification analyses per subgroup of PSP and FTD

**Fig. 5:**
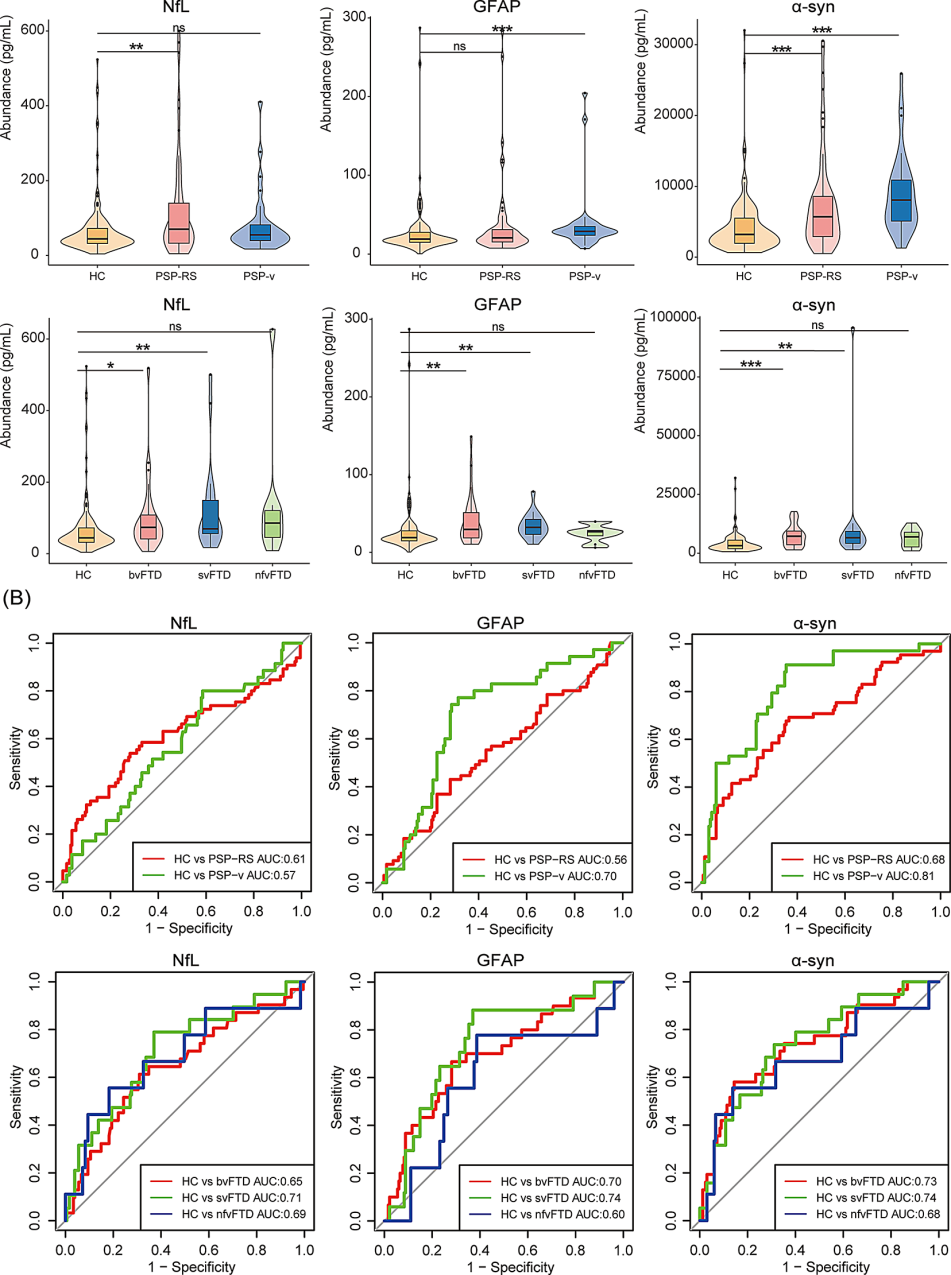
Plasma biomarkers of PSP and FTD subtypes (**A**) Comparison of blood biomarker levels across different PSP and FTD subtypes. Violin plots depict the median (horizontal bars) and interquartile range (IQR; hinges). *p*-values were assessed using the Wilcoxon test. *p < 0.05, **p < 0.01, ***p < 0.001. (**B**) Classification based on the area under the curve (AUC)

In this study, 100 patients with PSP were classified into the PSP-RS (*n* = 65) and PSP-variant (*n* = 35) subtypes, and the 76 patients with FTD was divided into the bvFTD (*n* = 31), nfvFTD (*n* = 9), and svFTD (*n* = 19) subtypes. We further explored the diagnostic performance of AD non-specific biomarkers, including GFAP, NFL, and α-synuclein, for detecting the above subtypes. As a result, we found that the α-synuclein level was the best for detecting PSP-v and bvFTD subtypes at cut-off values of 3957.3 pg/mL and 6705.0 pg/mL, achieving an AUC of 0.81 and 0.74, respectively, while the levels of GFAP and NFL presented good diagnostic performance in svFTD and nfvFTD at cut-off values of 22.32 pg/mL and 85.7 pg/mL, with an AUC of 0.74 and 0.69, respectively (Fig. 5).

### 3.7 Association between plasma biomarkers and Gray matter atrophy

**Fig. 6:**
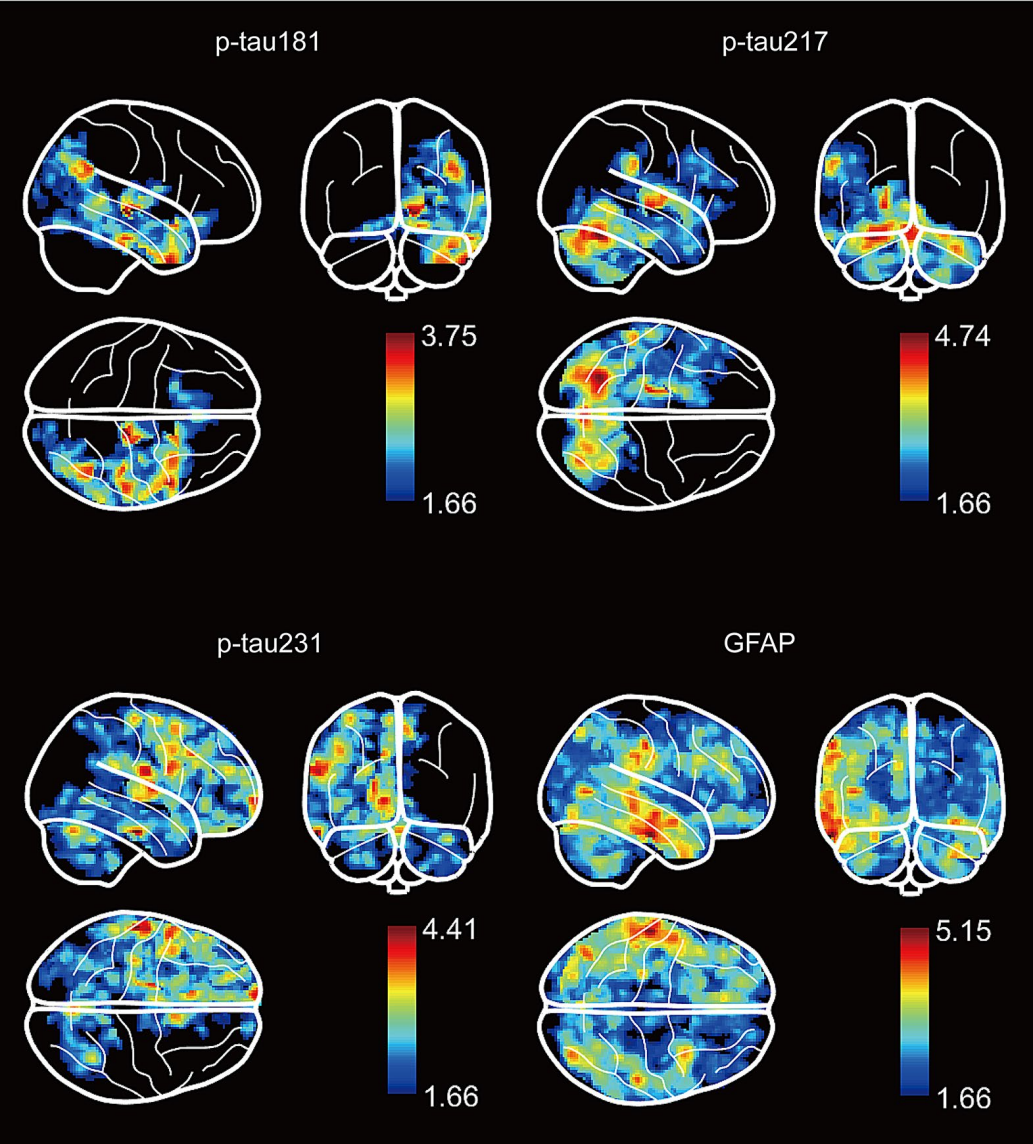
Results of the voxel-based analysis of gray matter volume and blood biomarker values. Results of the voxel-wise regression analyses show a significant relationship between brain atrophy measured on MRI and blood biomarkers after adjusting for sex and age. All results were adjusted for multiple comparisons using FWE (*q* < 0.05)

We constructed a GLM using MRI measurements as the dependent variable and blood biomarkers as independent variables. The results revealed that elevated levels of p-tau181 p-tau217, and GFAP were associated with reduced gray matter volume in the right, left and bilateral temporal lobe, respectively. And p-tau231 and GFAP were associated with reduced gray matter volume in the left and bilateral frontal lobe, respectively (*p* < 0.05) (Fig. 6).

### Association between longitudinal changes in plasma biomarkers and cognitive decline

**Fig. 7:**
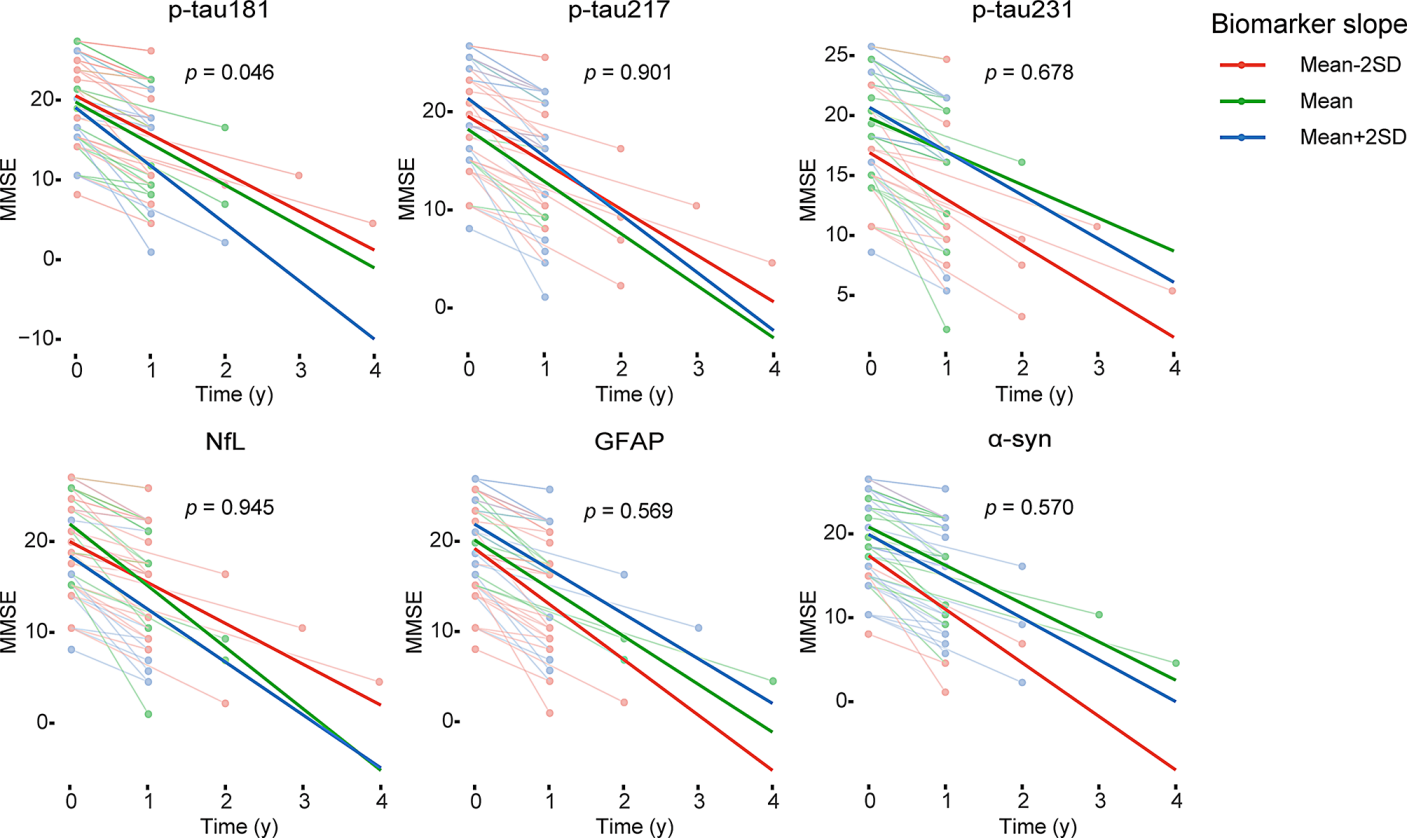
Association between longitudinal plasma biomarkers and MMSE. The x-axis shows the time from the first plasma biomarker samples. The model trajectories, shown as the mean slope and the mean ± 2 SD, were plotted from linear mixed effects models with the interaction between time and plasma biomarker slopes (derived from subject-level linear regression models) as an independent variable adjusting for sex and age

We further tested the association between longitudinal changes in plasma biomarker levels and longitudinal changes in cognition (as reflected by decreased MMSE scores). Longitudinal changes in p-tau181 levels over time were significantly associated with decreased MMSE scores (*p* = 0.046) (Fig. 7).

## Discussion

Evidence suggests that race and detection methods can affect blood biomarker levels in patients with AD and other types of dementia [35, 36]. However, there are few reports on the performance of these six blood biomarkers for the detection of AD in the Chinese population. To bridge this gap, we firstly conducted a large cross-sectional cohort study in the Chinese population, and simultaneously measured the levels of plasma p-tau181, p-tau217, p-tau231, NfL, GFAP, and α-synuclein using a novel single molecular immunity detection, where the α-synuclein level was reported for the first time. Based on our current result, most of biomarkers showed consistence changes across various dementia groups in the Chinese population compared to other populations, suggesting the potential applicability of plasma biomarkers for the diagnosis of AD in the Chinese population.

Increasing evidence suggests that plasma p-tau is the most promising diagnostic biomarkers for AD [37]. Patients with AD have elevated levels of plasma p-tau181, p-tau217, and p-tau231, which could distinguish AD from controls [6, 9, 11]. Until now, several studies have compared the AD diagnostic performance of p-tau isoforms with each other [13, 38, 39], while the efficiency was not consistent across different studies. In this study, p-tau217 and p-tau231 showed the best performance in AD diagnosis, with an AUC of 0.95 and 0.93, which was similar with Janelidze et al. and Yu et al. reported [13, 39]. Interestingly, we found that p-tau217, p-tau231 and p-tau181, have their advantages for the differential diagnosis of AD from PSP, FTD and DLB, respectively, among them, we first reported the p-tau181 performed best in the differential diagnosis between DLB and AD. With regard to the p-tau217 and p-tau231 for AD diagnosis from PSP and FTD, our finding is consistent with some previous studies simultaneously examining p-tau isoforms in neurodegenerative diseases. For example, Palmqvist et al. found that p-tau217 has the best performance in distinguishing AD and PSP [9], while Chouliaras et al. and Baiardi et al. showed that p-tau181 has the best identification performance for FTD [40, 41], which was not consistent with the current study. It is necessary to expand the number of non-AD dementia patients to verify our results. We also found that p-tau181, p-tau217, and p-tau231 were associated with Aβ pathology in the brain, and p-tau217 was associated with tau pathology in the brain, which is consistent with previous results [5, 42, 43], suggests that p-tau has a good predictive effect on AD-related pathology.

GFAP is cytoskeletal component of activated astrocytes whose levels increase in response to neuronal injury [44]. It has been previously reported that the level of plasma GFAP was elevated in AD and aMCI [2, 40, 41], which is consistent with our results. We also found that the diagnostic and differential diagnostic performance of GFAP was similar to that of plasma p-tau. Besides GFAP was significantly simultaneously related to CSF Aβ42/40 and p-tau, which is consistent with the results reported by Pereira et al. [45], and suggesting that GFAP can play a role in identifying AD-related pathologies. NfL is a cytoskeletal component of axons that is released into the blood during axonal degeneration [46]. We found that the plasma NfL level was higher in all dementia subtypes, but its diagnostic and differential diagnostic performance was poor, suggesting that it is unsuitable for diagnosis as a single indicator. However, our study further confirmed that these two biomarkers, prior to other biomarkers, could response to the disease severity. Furthermore, here, for the first time, we explored the value of plasma α-synuclein as a biomarker of dementia. Although total α-synuclein in the blood has several sources, the distribution of α-synuclein in the blood may provide a window into the α-synuclein in the brain of patients with neurodegenerative diseases and reflect changes in the degree of pathology [47]. We found that the plasma α-synuclein level was significantly higher in patients with AD and aMCI, and reached an AUC of 0.78 in terms of diagnostic efficacy. The good identification effect of α-synuclein on AD and aMCI, as well as its associations with CSF Aβ42/40, proved that α-synuclein also can be used as a potential biomarker for AD. Notably, there have been no reports on plasma α-synuclein in clinical subgroups of PSP and FTD. Our research showed that α-synuclein has good discrimination ability on PSPv and bv FTD, which confirmed that the α-synuclein pathology may exist in common neurodegenerative dementias.

Notably, Studies highlighted that AD-copathology is common in dementia with Lewy bodies (DLB), affecting nearly half of cases. This co-pathology accelerates cognitive decline, worsening prognosis, and often complicating diagnosis [48, 49]. This is consistent with our results showing that some non-AD patients have AD co-pathology. The co-occurrence of AD pathology in DLB impacts biomarker levels and requires integrated biomarker approaches for accurate diagnosis and prognosis assessment. Our results showed that plasma p-tau217, NfL, and GFAP were valuable in detecting AD-copathology in non-AD patients, aiding in the identification of mixed pathology cases.

This study also revealed that longitudinal changes in p-tau181 were associated with changes in cognition, suggesting that plasma p-tau181 has an advantage in monitoring dynamic disease progression because it continues to increase during disease development, where the rate of increase correlates with cognitive decline. Our results support the use of plasma p-tau181 as a surrogate outcome biomarker in intervention trials and for tracking disease progression in clinical practice. However, these results are inconsistent with the findings of Ashton et al., which showed that longitudinal changes in p-tau217, but not p-tau181, were associated with cognitive changes [14]. The difference may be due to the small sample size of our longitudinal cohort; thus, a larger sample size is needed to verify our results in the future, it is also possible that these differences are attributable to ethnic variation. The LOD and LOQ results of SMID detection based on AST-Sc-Lite showed that it had high sensitivity to plasma biomarkers, suggesting that this method can be used for the detection of trace components in plasma like SIMOA.

Several studies have investigated AD plasma biomarkers in Chinese populations, emphasizing the diagnostic utility of plasma biomarkers such as p-tau, NfL, and GFAP [15, 50–53]. The findings reveal that elevated levels of p-tau and GFAP are linked to AD pathology, astrocyte reactivity, and neurodegeneration [54], while p-tau181 and NfL correlates with disease progression [55]. Our study builds on these findings by further validating the role of these plasma biomarkers specifically in the Chinese population. By analyzing a broad range of AD biomarkers, we demonstrate the practicality and clinical relevance of plasma biomarkers in the context of diverse Chinese cohorts.

The LOD and LOQ results of SMID detection based on AST-Sc-Lite showed that it has high sensitivity for biomarkers in plasma, suggesting that this method has the potential to be used for the plasma biomarkers detection like SIMOA. The detection results of SMID detection based on AST-Sc-Lite show strong consistency with those of SIMOA method and MSD method. In addition, the changes in biomarker levels found in our study were consistent with those in previous studies using the classical SIMOA method, further demonstrating the effectiveness of this new method.

However, the limitation of this study is that we did not have CSF or PET confirmation of diagnosis for all our participants and therefore it is likely some of the clinical diagnoses arise from mixed pathologies or alternative pathologies; and SIMOA tests were not performed on all indicators in the same cohort to verify the consistency of the results of the new and the classical method. In addition, data reproducibility and preanalytical confounders, such as the release of α-synuclein from blood cells are main hampering issues in α-synuclein detection [56]. In our study, we followed a strict sample collection standard operating procedure (SOP), and our samples were not subjected to freeze-thaw cycles, thus minimizing this risk. However, further studies with larger sample sizes are necessary to establish alpha-synuclein’s validity as a biomarker, particularly in plasma, where contamination risks must be carefully controlled to avoid confounding results.

## Conclusions

This study validates the practicality of blood biomarkers in the Chinese Han population using a novel single molecule immune detection method. Through the comparison study for several biomarkers, we found the plasma p-tau217 was the most effective biomarker in AD diagnosis, and p-tau217, p-tau231 and p-tau181 showed the best performance for differential diagnosis of AD from PSP, FTD and DLB respectively, GFAP presented the most significant correlations with AD pathology, and gray matter volume reduction of frontal and temporal lobe. while p-tau181 can best reflect the dynamic progression of AD.

## Electronic supplementary material

Below is the link to the electronic supplementary material.

Supplementary Material 1

## Data Availability

The data that support the findings of this study are available from the authors.

## Abbreviations

AD: Alzheimer’s disease
Aβ: Amyloid beta
PET: Positron emission tomography
CSF: Cerebrospinal fluid
MCI: Mild cognitive impairment
aMCI: Amnesic MCI
FTD: Frontotemporal lobar degeneration
PSP: Progressive supranuclear palsy
DLB: Dementia with Lewy bodies
NfL: Neurofibrillary light chain
GFAP: Glial fibrillary acidic protein
IMR: Immunomagnetic reduction
ELIZA: Enzyme-linked immunosorbent assay
SIMOA: Single-molecule assay
FWHM: Full width at half maximum
GLM: General linear model
TIV: Total intracranial volume
MMSE: MINI Mental State Examination
MoCA: Montreal Cognitive Assessment
ROC: Receiver operating characteristic
AUC: Area under the curve
CDR: Clinical Dementia Rating
