# Supplementary material for: Evaluating the diagnostic performance of six plasma biomarkers for Alzheimer’s disease and other neurodegenerative dementias in a large Chinese cohort
Source: Alzheimers Res Ther. 2025 Apr 3;17:71. doi: 10.1186/s13195-025-01712-y (PMC11969745; doi:10.1186/s13195-025-01712-y)
Supplement: Supplementary file 1 — Supplementary Material 1 [file 13195_2025_1712_MOESM1_ESM.docx]

**Supplementary Materials**

***Section 1. Supplementary Methods***

**Schematic illustration of Single Molecule Immune Detection (SMID) assay**


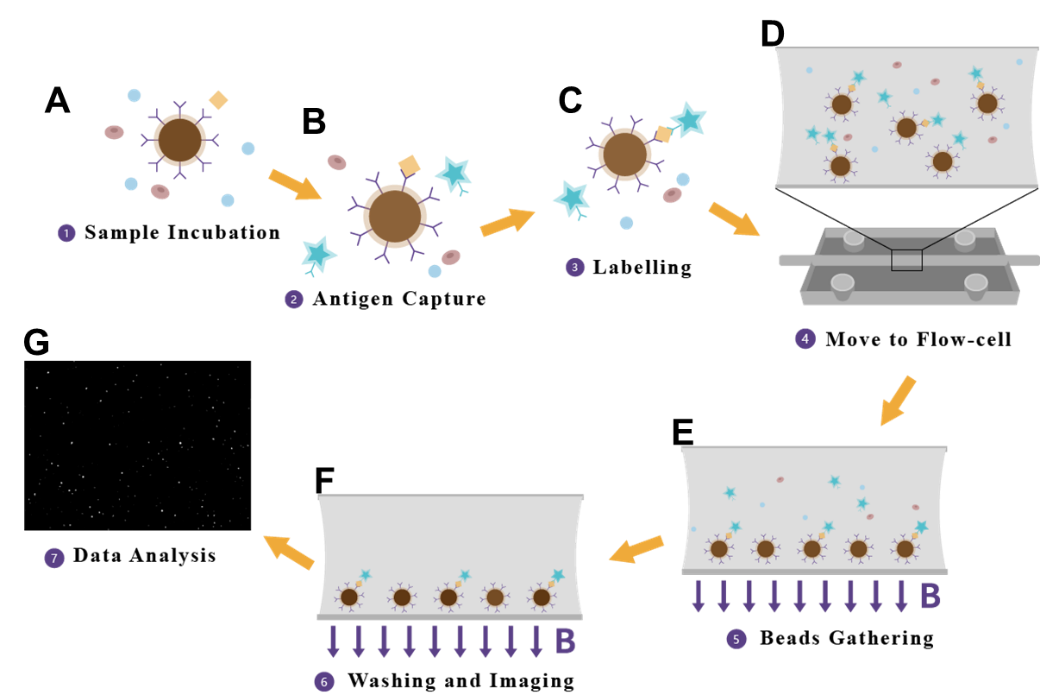


**Supplementary Figure 1.** Schematic illustration of Single Molecule Immune Detection (SMID) assay: (A)-(B) Blood sample is firstly incubated with capture-antibody-coated magnetic beads. Target molecules in sample are captured the beads. (C) By directly labeling with homemade single molecule imaging fluorophore labelled with detection antibody, which enables single particle imaging in the automatic SMID system, (D) the reaction mixture is moved into a glass flow-cell channel. (E) Magnetic beads are absorbed onto bottom surface of flow-cell to wash away unbonded fluorophore. The guarantee a stable measurement result, the absorbed beads are well controlled by precise control of magnetic field intensity, magnetic beads concentration and washing flow rate. The gathered magnetic beads generate single-layer random monodispersed beads array on the surface, allowing single fluorophore imaging for following analysis, whose number can be well controlled within 1.2%. (F) (G) The fluorescence image of fluorophore is capture by an integrated camera after the fluorophore are excited by a LED light source.

**Building of standard curves for plasma Aβ1-40, Aβ1-42，p-Tau181, p-Tau217, p-Tau231, NFL , GFAP and α-synuclein measuring**

Standards of Aβ1-40, Aβ1-42, p-Tau181, p-Tau217, p-Tau231, NFL, GFAP and α-synuclein are diluted with commercial plasma matrix with biomarkers removed as **Supplementary Table 1**. Each diluted sample was measured three times as following steps on AST-Sc-Lite (A fully-auto single-molecule detection machine supplied by Suzhou AstraBio technology Co., Ltd.): (i) Load 25 μL sample into an incubation tube and add 25 μL Reagent 1 (mainly comprised 0.1 mg/mL magnetic beads coated with capture antibodies and protecting reagents), followed by a quick mixing automatically by the machine. (ii) After a 6-minutes incubation, 10 μL Reagent 2 (comprised of detection antibodies labeled with single-molecule imagine fluorophores) was added, mixed, and incubated for 4 minutes under 40°C. (iii) Magnetic beads in the mixtures were absorbed onto the surface of the channel in the flow cell by a permanent magnet. Unlabeled fluorophores were removed by a gentle washing flow of wash buffer and fluorescent images were then taken with an integrated fluorescent microscope. (IV) Standard curves for each plasma biomarkers are built with home-build fitting software (**Supplementary Fig. 2**). Typical fluorescent images of fluorophore labelled magnetic beads array by p-Tau217 was shown in **Supplementary Figure 3**.

**Supplementary Table 1.** Biomarker diluent ladder for standard curve building.

| Aβ40 | Aβ42 | p-Tau181 | p-Tau217 | p-Tau231 | NFL | GFAP | α-syn |
| --- | --- | --- | --- | --- | --- | --- | --- |
| pg/mL | | | | | | | |
| 0 | 0 | 0 | 0 | 0 | 0 | 0 | 0 |
| 5.65 | 5.08 | 2.82 | 0.56 | 0.28 | 5.08 | 5.08 | 16.94 |
| 16.94 | 15.24 | 8.47 | 1.69 | 0.85 | 15.24 | 15.24 | 50.81 |
| 50.81 | 45.72 | 25.4 | 5.08 | 2.54 | 45.72 | 45.72 | 152.42 |
| 152.42 | 137.17 | 76.21 | 15.24 | 7.62 | 137.17 | 137.17 | 457.25 |
| 457.25 | 411.52 | 228.62 | 45.72 | 22.86 | 411.52 | 411.52 | 1371.74 |
| 1371.74 | 1234.57 | 685.87 | 137.17 | 68.59 | 1234.57 | 1234.57 | 4115.23 |
| 4115.23 | 3703.7 | 2057.61 | 411.52 | 205.76 | 3703.7 | 3703.7 | 12345.7 |
| 12345.7 | 11111.1 | 6172.84 | 1234.57 | 617.28 | 11111.1 | 11111.1 | 37037 |
| 37037 | 33333.3 | 18518.5 | 3703.7 | 1851.85 | 33333.3 | 33333.3 | 111111 |
| 111111 | 100000 | 55555.6 | 11111.1 | 5555 | 100000 | 100000 | 333333 |

**
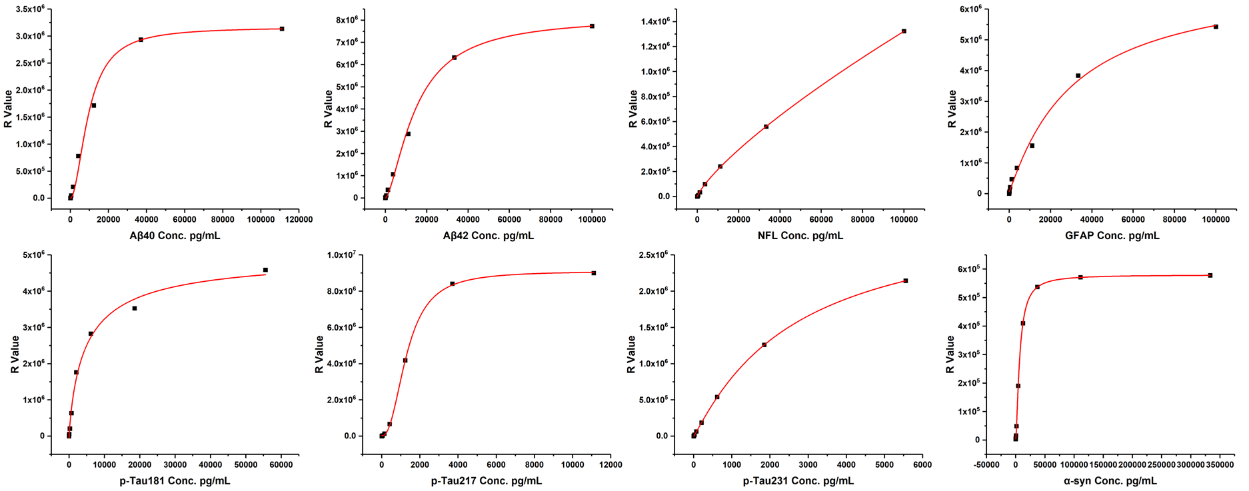
**

**Supplementary Figure 2.** Fitted standard curves for each biomarker.


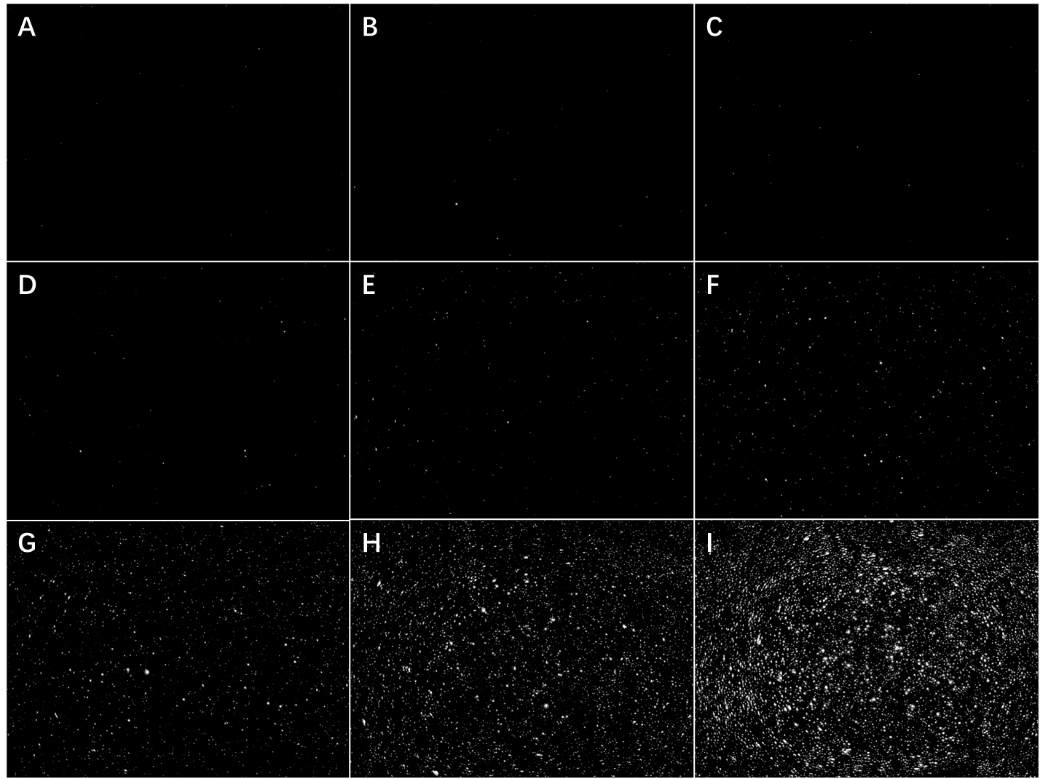


**Supplementary Figure 3.** A series typical fluorescent image of fluorophore labelled magnetic beads array by p-Tau217 with concentration of 0, (A) 0.56, (B) 1.69, (C)5.08, (D)15.24, (E)45.72, (F)137.17, (G)411.52, (H)1234.57, (I)3703.7 pg/mL.

**Limit of detection (LOD) and limit of quantification (LOQ) of AST-Sc-Lite**

The LOD and LOQ results for 10 min incubation are presented in **Supplementary Table 2**. The LOD was determined by *2SD_blank_/S*. In which, *SD_blank_* is standard deviation 20 repeats measurement of dilution buffer for each biomarker and *S* is slope of linear regression formula near blank. To determine LOQ, a series of samples with varying concentrations of each biomarker were prepared. These samples were then measured five times each. The concentration at which the coefficient of variation (CV%) of the measurements was approximately 20% was identified as LOQ.

**Supplementary Table 2.** LOD and LOQ results.

|  | **LOD** | **LOQ** |
| --- | --- | --- |
| **P-tau181** | 0.12 pg/mL | 0.319 pg/mL |
| **P-tau217** | 0.096 pg/mL | 0.252 pg/mL |
| **P-tau231** | 0.53 pg/mL | 0.87 pg/mL |
| **GFAP** | 0.61 pg/mL | 1.30 pg/mL |
| **NfL** | 0.95 pg/mL | 1.68 pg/mL |
| **α-syn** | 15.8 pg/mL | 53.3 pg/mL |

**Assay comparison**

Comparison of clinical sample results for the SMID (AST-Sc-Lite) with the SIMOA (Quanterix) and the MSD (MESO QuickPlex) are shown in **Supplementary Table 3,** **Supplementary Figure 4** and **5**, respectively. NfL levels in 23 plasma samples were compared between the SMID (AST-Sc-Lite) and the SIMOA (Quanterix). p-tau217 levels in 24 plasma samples were compared between the SMID (AST-Sc-Lite) and the MSD (MESO QuickPlex). The results showed that SMID (AST-Sc-Lite) had a strong correlation with the SIMOA (Quanterix) test (R^2^ = 0.99) and the MSD (MESO QuickPlex) test results (R^2^ = 0.95).

**Supplementary Table 3.** NfL concentrations in clinical plasma samples using SMID (AST-Sc-Lite) and SIMOA (Quanterix).

| **Num.** | **Quanterix pg/mL** | **AST-Sc-Lite pg/mL** |
| --- | --- | --- |
| 1 | 10.62 | 10.31 |
| 2 | 5.77 | 7.5 |
| 3 | 61.94 | 55.32 |
| 4 | 8.7 | 13.46 |
| 5 | 54.95 | 58.25 |
| 6 | 7.33 | 22.99 |
| 7 | 432.29 | 460.85 |
| 8 | 14.60 | 12.97 |
| 9 | 26.49 | 25.66 |
| 10 | 97.83 | 87.67 |
| 11 | 17.16 | 26.98 |
| 12 | 37.7 | 40.91 |
| 13 | 101.16 | 108.15 |
| 14 | 53.35 | 97.2 |
| 15 | 28.35 | 35.83 |
| 16 | 195.76 | 173.29 |
| 17 | 363.35 | 378.99 |
| 18 | 21.95 | 38.36 |
| 19 | 318.24 | 316.81 |
| 20 | 341.92 | 392.52 |
| 21 | 11.39 | 16.92 |
| 22 | 65.84 | 40.78 |
| 23 | 47.06 | 37.56 |

**
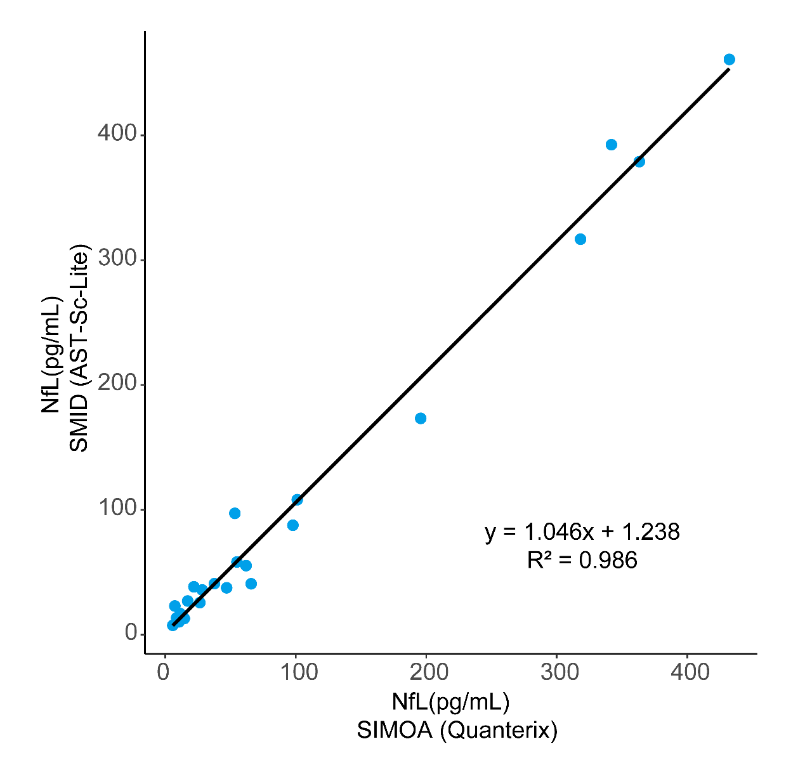
**

**Supplementary Figure 4.** Correlations of measured NfL concentrations in clinical plasma samples using SMID (AST-Sc-Lite) and SIMOA (Quanterix). A total of 23 samples were tested. Statistical analysis (R = 0.986) demonstrated the strong correlation between both data sets.


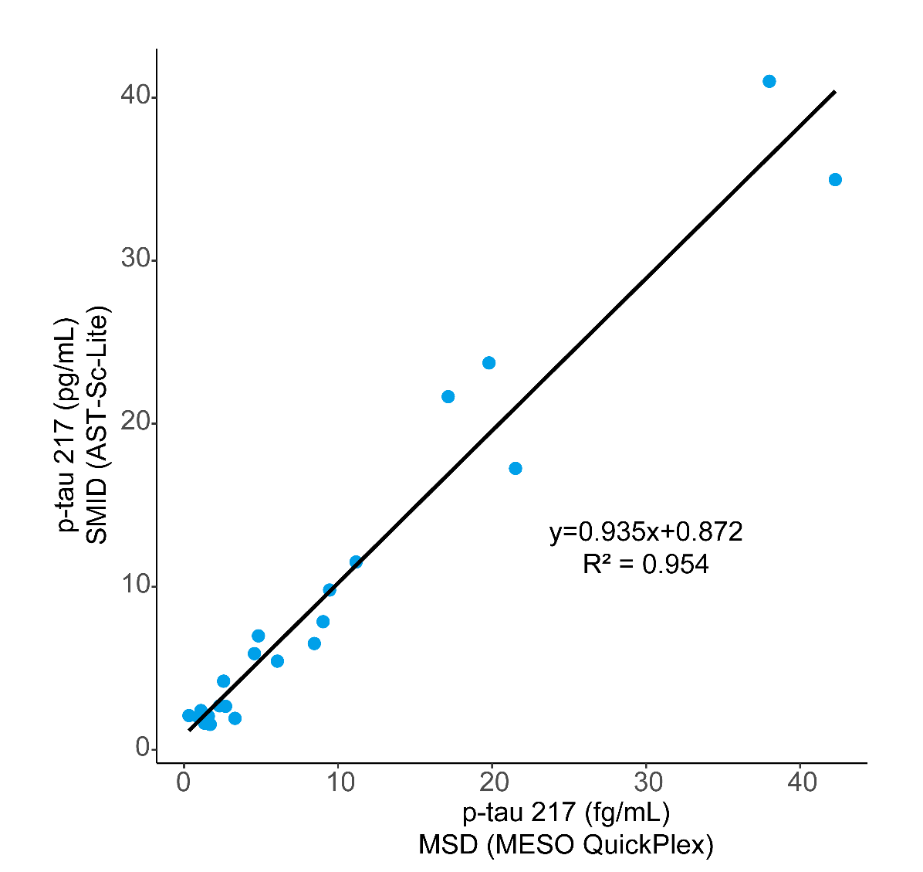


**Supplementary Figure 5.** Correlations of measured p-tau 217 concentrations in clinical plasma samples using SMID (AST-Sc-Lite) and MSD (MESO QuickPlex). A total of 24 samples were tested. Statistical analysis (R = 0.954) demonstrated the strong correlation between both data sets.


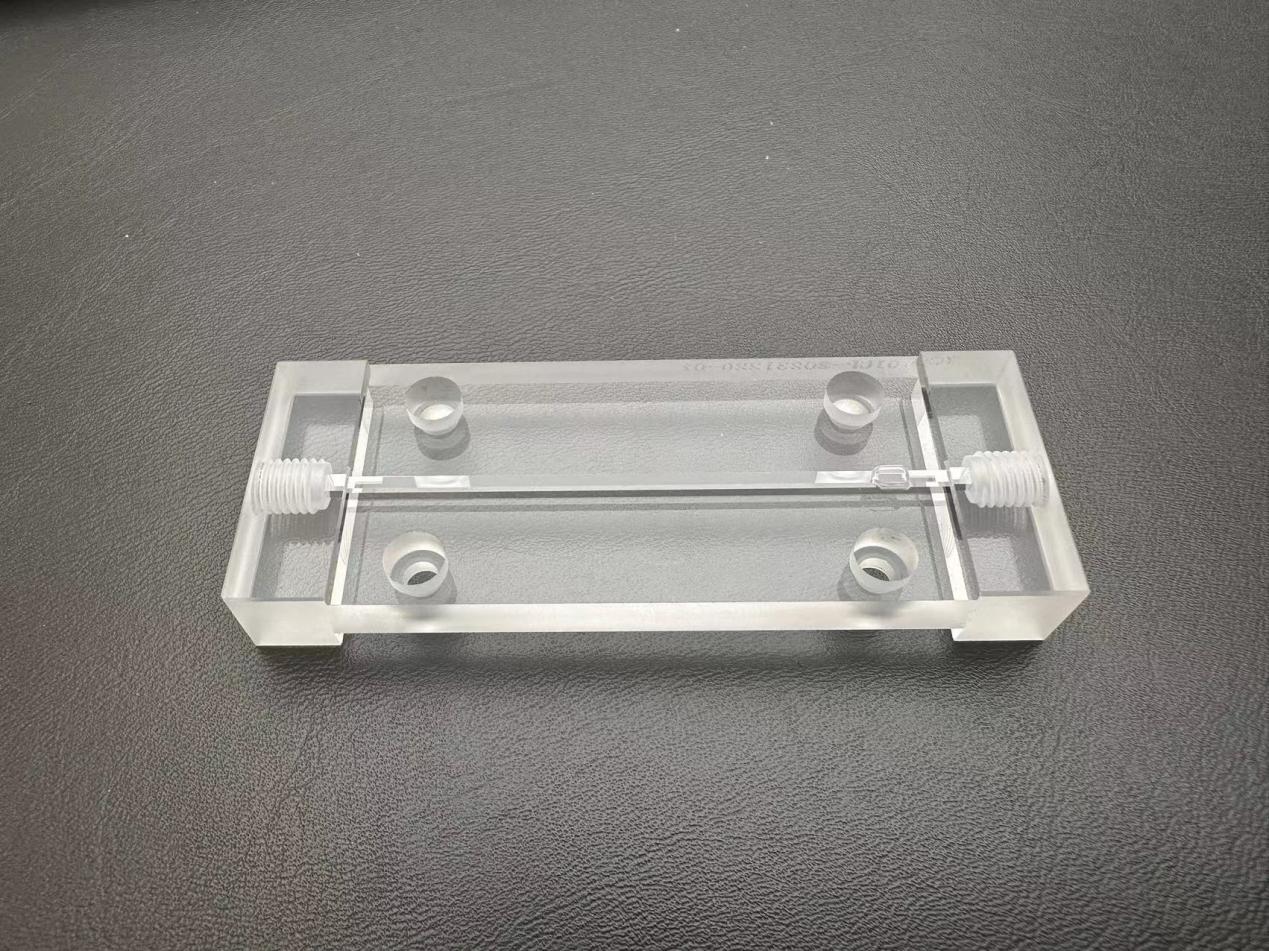


**Supplementary Figure 6.** Image of flow-cell in Astra System for randomly monodispersed single layer magnetic beads array generation.


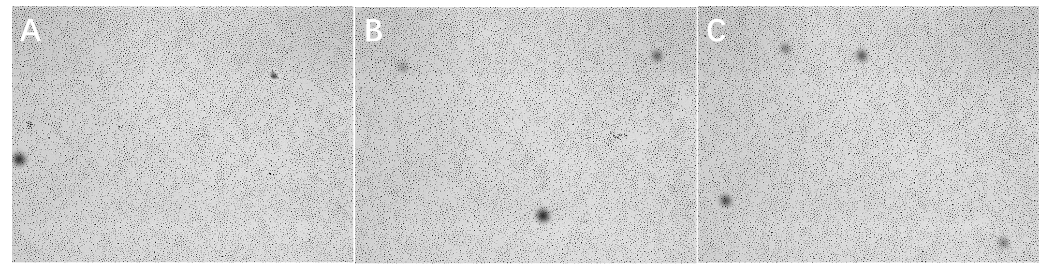


**Supplementary Figure 7.** A typical image under bright-field of 3 repeats of single-layer magnetic beads array.

***Section 2. Plasma Biomarker Comparisons Across AD Pathology in Subgroups***

**
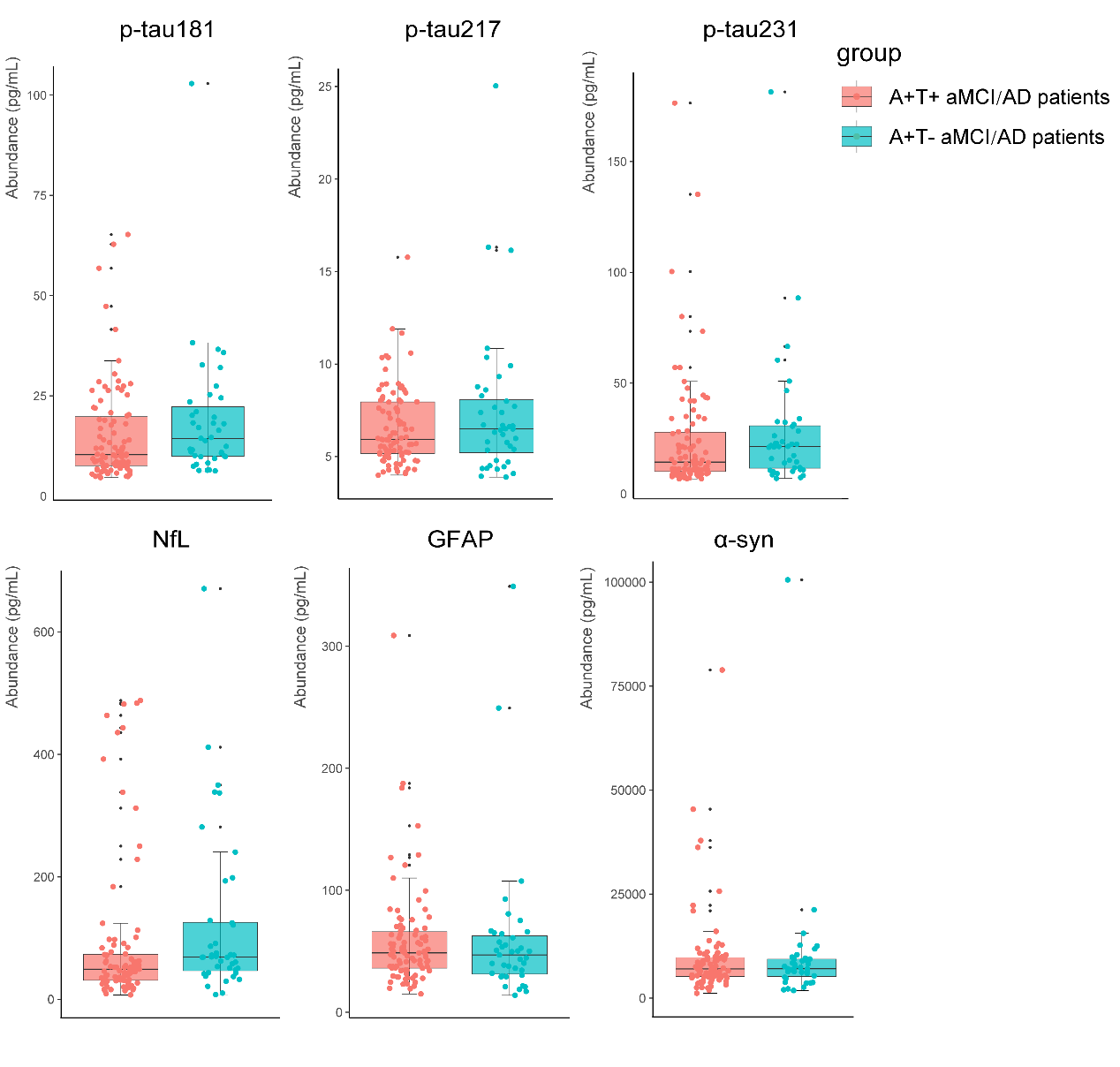
**

**Supplementary Figure 8.** Plasma biomarkers in Α+T+ versus A+T- group within aMCI and AD patients . Box plots depict the median (horizontal bar) and interquartile range (IQR; hinges). P-values were assessed using the Wilcoxon test. **p* < 0.05, ***p* < 0.01, ****p* < 0.001.


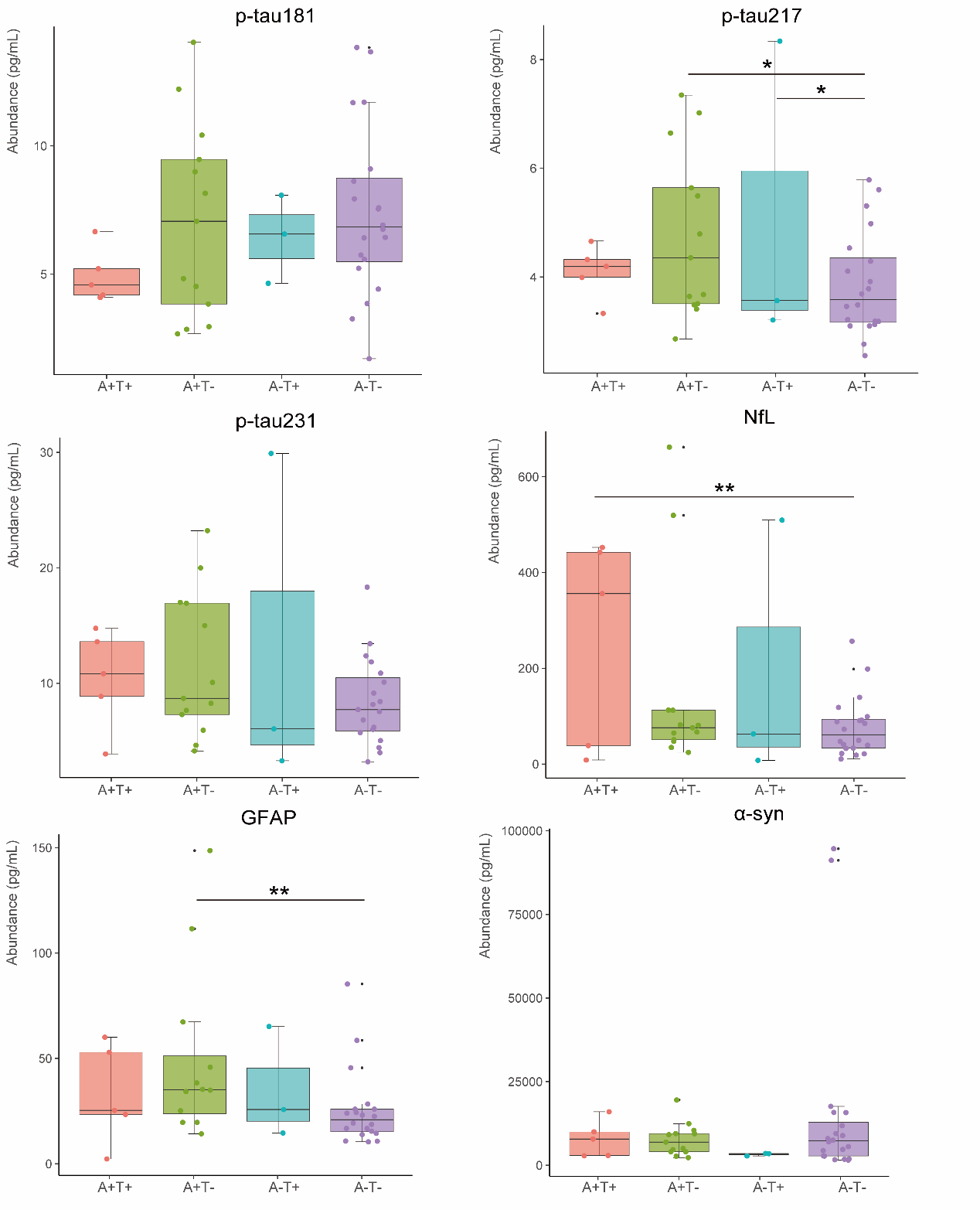


**Supplementary Figure 9.** Plasma biomarkers in A+T+, A+T-, A-T+ and A-T- groups within non-AD (FTD and DLB) patients. Box plots depict the median (horizontal bar) and interquartile range (IQR; hinges). P-values were assessed using the Wilcoxon test. **p* < 0.05, ***p* < 0.01, ****p* < 0.001.

***Section 3. Plasma Biomarker Comparisons in Different Levels of Cognitive Impairment***
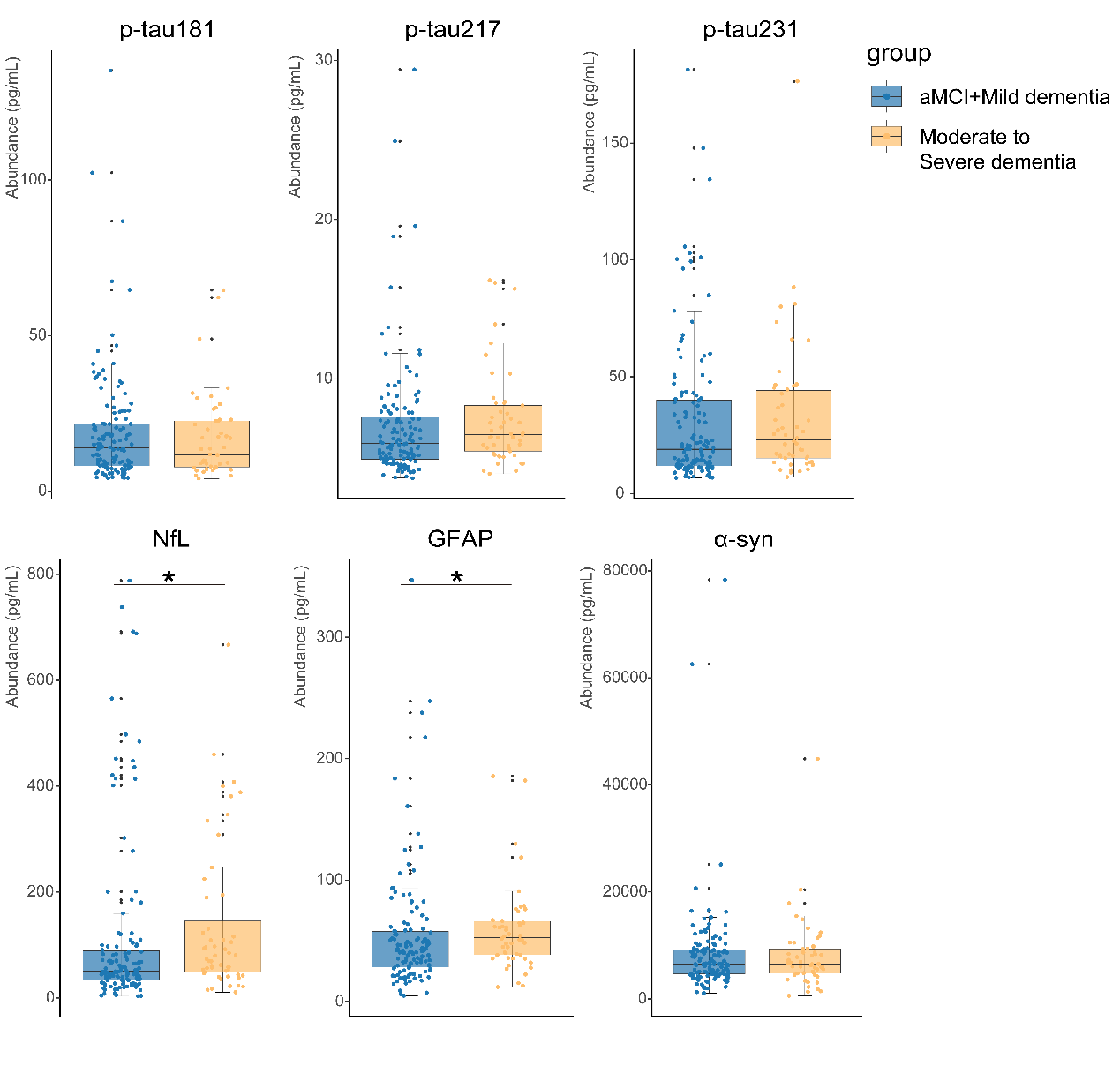


**Supplementary Figure 10.** Plasma biomarkers in patients with dementia with different levels of cognitive impairment. Box plots depict the median (horizontal bar) and interquartile range (IQR; hinges). P-values were assessed using the Wilcoxon test. **p* < 0.05, ***p* < 0.01, ****p* < 0.001.
